# Unusual presentation of metastatic melanoma

**DOI:** 10.1093/jscr/rjae429

**Published:** 2024-06-26

**Authors:** Anthony La, Armand Asarian, Philip Xiao

**Affiliations:** Department of Medicine, St. George’s University School of Medicine, West Indies, Grenada; Department of Surgery, The Brooklyn Hospital Center, Icahn School of Medicine at Mount Sinai, Brooklyn, NY 11201, United States; Department of Pathology, The Brooklyn Hospital Center, Icahn School of Medicine at Mount Sinai, Brooklyn, NY 11201, United States

**Keywords:** melanoma, metastatic, urinary, bladder, hematuria

## Abstract

Urinary bladder with concurrent colonic melanoma is an exceptionally uncommon occurrence, posing a diagnostic challenge for clinicians. While rare, it warrants consideration as a potential differential diagnosis, particularly in patients without a history of melanoma who present with persistent hematuria due to its aggressive nature. We present a case of a 55-year-old female with malignant melanoma involving the colon and urinary bladder presenting with hematuria. Given the scarcity of cases and variability in clinical management approaches, there is a pressing need for research efforts to establish standardized protocols and conduct trials to guide clinical practice in this rare entity.

## Introduction

Melanoma is the most common skin cancer-related deaths dividing into categories including skin, eyes, and mucosal melanoma. Of the skin, superficial spreading melanoma, nodular melanoma, lentigo maligna melanoma, and acral lentiginous melanoma are considered the four main subtypes [1]. Melanoma of the eyes encompasses choroidal, conjunctival, and iris melanoma. Mucosal melanoma involves mucosal linings of head, neck, vagina, gastrointestinal regions, and urinary tract.

We present a rare case of malignant melanoma in a 55-year-old female affecting the urinary bladder and colon. Mucosal melanomas comprise only 1% of all melanoma cases globally, with primary melanoma of the urinary tract presenting less than 4% of mucosal melanomas [2].

## Case presentation

A 55-year-old female presenting with 6 months hematuria who underwent a cystoscopy and transurethral resection of bladder tumor (TURBT) shows both superficial and deep malignant melanoma involving the urinary bladder. Colonoscopy reveals an ulcerated lesion in ascending colon.

## Pathological findings

**Figure 1:**
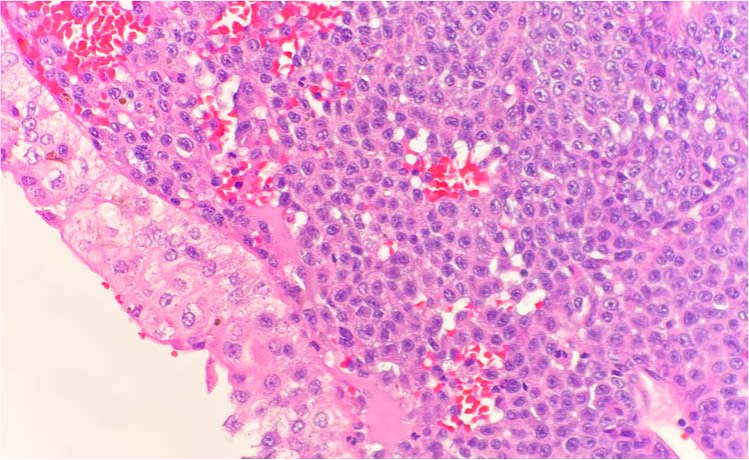
Microscopic examination reveals sheets of round cell beneath unremarkable urothelium epithelium. H&E stain 40×.

**Figure 2:**
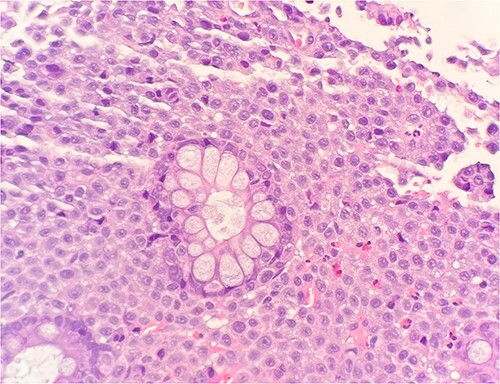
Microscopic examination reveals sheets of round cell between unremarkable colonic crypts. H&E stain 40×.

Microscopic examination shows extensive involvement of the bladder wall by sheets of small round blue tumor cells showing cleaved and irregular nuclear contours and conspicuous nucleoli. Mitotic activity is frequent and majority of cells show scant amphophilic cytoplasm with few foci showing more abundant bubbly cytoplasm and others with clear foamy cytoplasm (Fig. 1). A rare foci of finely granular brown pigment representing melanin and moderate cytological atypia in a small area of surface urothelium. Similar morphological features are seen in colonic biopsy specimens (Fig. 2).

**Figure 3:**
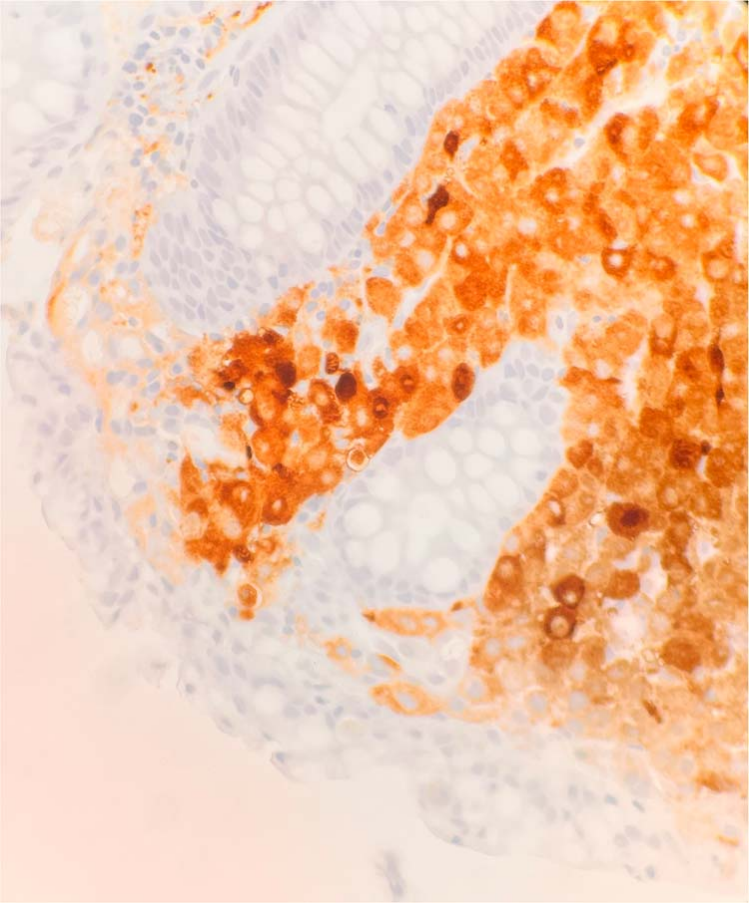
Microscopic examination reveals sheets of round are positive for S100. IHC stain 40×.

Immunohistochemical stains demonstrate the tumor cells to be strongly positive for vimentin, pankeratin, CK7, CK20, chromogranin, synaptophysin, CD45, and focally positive for CD56. Additionally, there were strongly and diffusely positive findings for S100 (Fig. 3), SOX10, and PRAME. Ki67 shows increased proliferative activity at estimated 25–30%. CD99 shows weak and patchy membranous staining while CK-PAN, p63, desmin, and smooth muscle actin are negative. The described morphology and immunoprofile are consistent with malignant melanoma.

Colonoscopy a few months later revealed multiple polyps from transverse, descending, and sigmoid colon. Microscopic examination reveals metastatic malignant melanoma with an unknown primary origin. Immunostains were positive for vimentin, CD117, and S100. Immunostains were strongly positive for SOX10 and PRAME (preferentially expressed antigen of melanoma); while negative for BRAF, VE1, NRAS, Q61L, and DOG1. Status post cystoscopy and TURBT, a follow up PET scan shows interval resolution of previously seen subcentimeter nodular density within the bladder. Patient is now on an immunotherapy regime.

## Discussion

Melanoma involving the urinary bladder and colon is exceptionally rare among all reported cases of melanoma; especially primary bladder melanoma accounting for <50 cases reported worldwide [3]. Melanoma of the urinary bladder mainly affects those aged between 44 and 81 years old without gender preference or a clear consensus for etiological association [2, 3]. Almost all reported cases featured hematuria as the primary clinical feature. Although hematuria consists of a wide range of differentials with melanoma being low on the list or overlooked, its consideration is important due to its high mortality tendencies. Increased suspicion should be prompted in patients with known melanoma presenting with persistent hematuria considering most bladder melanomas are presented metastatically from another site. Isolated bladder melanomas upon presentation should also consider further work up for metastasis given primary origin bladder melanoma is exceedingly rare. As with many melanoma, presence of metastatic lesions, size and depth of invasion are prognostic of the outcome but no standard protocol for staging or treatments exists for this disease yet [2].

Many cytological features seen in melanoma of the bladder show cells with pleomorphic individual/clusters nuclei, spindle cells, cytoplasmic melanin pigment, and prominent eosinophilic nucleoli [3]. Urothelium is usually spared as evident by our patient’s presentation with only a small section of surface urothelium showing cytologic atypia [4]. Melanoma of the urinary bladder stains positively for S100, HMB45, SOX10, and negatively for keratin and vimentin [3] consistent with our patient’s positively stained immunohistochemistry. Molecular studies of urinary tract melanoma are finite and still require extensive research, although it has been shown that a small subgroup of primary urinary tract melanomas (~14%) is characterized by c-kit mutation. In contrast, the cutaneous variant known for BRAF mutations is rarely observed in urinary tract melanomas [5].

Due to the infrequency of these cases, there have been multiple reported therapeutic approaches including TURBT, partial/radical cystectomy, radiotherapy, chemotherapy, local excision, wide resection surgery with lymph node dissection, interferon α, and intravesical instillation of Bacillus-Calmetter–Guerin without a clear first-line consensus of what is the optimal management [2, 3]. The patient underwent cystoscopy, TURBT, followed up with ipilimumab/nivolumab immunotherapy with reports of significant decrease in quality of life likely due to transfusions.

Despite a lack of standard protocol for this condition, medical advancements throughout the decades allow us to have multiple therapeutic approaches when faced with an unknown entity. Numerous approaches have been reported and consideration for research should look into performing trials and standardizing a protocol best suited for melanoma of the bladder.

## Conclusion

The presence of melanoma in the urinary bladder and colon at the same time is exceedingly rare and the majority of practicing physicians may not ever encounter it. However, it should be noted as a differential in patients presenting with persistent hematuria and ruled out for its aggressive mortality potential. Due to its scarce incidence and lack of uniformed approach among clinicians, consideration for research should focus on performing trials and standardizing a protocol.
